# The impact of Transcranial Magnetic Stimulation (TMS) on seizure course in people with and without epilepsy

**DOI:** 10.1016/j.cnp.2022.05.005

**Published:** 2022-06-13

**Authors:** Serena Pang, Sasha D'Ambrosio, Giulia Battaglia, Diego Jiménez-Jiménez, Marco Perulli, Katri Silvennoinen, Sara Zagaglia, Sanjay M. Sisodiya, Simona Balestrini

**Affiliations:** a Department of Clinical and Experimental Epilepsy, UCL Queen Square Institute of Neurology, London, and Epilepsy Society, Chalfont-St-Peter, Bucks, UK; b School of Medicine, Universidad San Francisco de Quito, Quito, Ecuador; c Child Neurology and Psychiatry Unit, Fondazione Policlinico Universitario Agostino Gemelli IRCCS, Rome, Italy; d Department of Neuroscience, Catholic University Of The Sacred Heart, Rome, Italy; e Neuroscience Department, Children's Hospital Meyer-University of Florence, Florence, Italy

**Keywords:** AHC, Alternating Hemiplegia of Childhood, ASM, Anti-Seizure Medication, CNS, Central Nervous System, E:I Ratio, Excitation: Inhibition Ratio, EEG, Electroencephalography, EMG, Electromyography, EOG, Electrooculography, FDI, First Dorsal Interosseus, HD-EEG, High-Density EEG, ICF, Intracortical Facilitation, LICI, Long Interval Cortical Inhibition, MEP, Motor Evoked Potential, MRI, Magnetic Resonance Imaging, MSO, Maximum Stimulator Output, MT, Motor Threshold, ppTMS, Paired-pulse TMS, PWE, People with Epilepsy, QoL, Quality of Life, RMT, Resting Motor Threshold, rTMS, Repetitive TMS, SCORE, Standardized Computer-based Organized Reporting of EEG, SICI, Short-Interval Intracortical Inhibition, spTMS, Single-pulse TMS, TEP, TMS-evoked Potential, TMS, Transcranial Magnetic Stimulation, TMS, Safety, Single pulse, Paired pulse, Alternating hemiplegia of childhood

## Abstract

**Objective:**

To elucidate the effects of single and paired-pulse TMS on seizure activity at electrographic and clinical levels in people with and without epilepsy.

**Methods:**

A cohort of 35 people with epilepsy, two people with alternating hemiplegia of childhood (AHC) with no epilepsy, and 16 healthy individuals underwent single or paired-pulse TMS combined with EEG. Clinical records and subject interviews were used to examine seizure frequency four weeks before and after TMS.

**Results:**

There were no significant differences in seizure frequency in any subject after TMS exposure. There was no occurrence of seizures in healthy individuals, and no worsening of hemiplegic attacks in people with AHC.

**Conclusions:**

No significant changes in seizure activity were found before or after TMS.

**Significance:**

This study adds evidence on the safety of TMS in people with and without epilepsy with follow-up of four weeks after TMS.

## Highlights

• Previous evidence on risk of TMS-induced seizures is anecdotal.
• There was no evidence that TMS caused changes in epileptiform activity.
• No seizures were induced by TMS in healthy subjects.

## 1 Introduction

Epilepsy is a neurological disorder affecting over 70 million people worldwide (Fisher et al., 2014, Moshé et al., 2015). People with epilepsy (PWE) experience epileptic seizures, clinically defined as transitory episodes of abnormal and excessive action potentials firing simultaneously (Fisher et al., 2005, Falco-Walter et al., 2018). Epilepsy can detrimentally impair cognition and decrease quality of life (QoL), with up to 30% of individuals developing drug-resistant epilepsy (DRE) (Engel, 2011). Antiseizure medications (ASM) are a key treatment for epilepsy (Duncan et al., 2006, Perucca and Tomson, 2011). Despite the advent of new ASM within the last few decades, there has been no improvement in treatment response overall (Brodie, 2017, Chen et al., 2018). Further research is required to investigate mechanisms of epileptogenesis and new treatment strategies, and new research tools are needed.

Transcranial Magnetic Stimulation (TMS) is a non-invasive brain stimulation technique designed to evaluate cortical functions and brain dynamics. This method was implemented in the mid-1980’s and has rapidly evolved from a simple technique for studying motor pathways to a cutting-edge neurophysiological methodology (Barker et al., 1985). Recently, it has been used in several branches of the neurosciences for investigating physiological and pathological mechanisms, for example in disorders of consciousness, schizophrenia, and epilepsy (Ferrarelli et al., 2012, Casarotto et al., 2016, Kimiskidis et al., 2017, Tremblay et al., 2019). TMS is administered to the human cortex *in vivo*. It induces a focal electric field which depolarises axonal membrane thereby probing brain excitability and effective connectivity (Rothwell et al., 1987, Kobayashi and Pascual-Leone, 2003, Massimini et al., 2005). Stimulation configurations can vary, including single pulse TMS (spTMS) where a single pulse is administered once every few seconds (<0.5 Hz), paired pulse (ppTMS) with two sequential pulses delivered within a short interval and repeated every few seconds (<0.5 Hz), and repetitive TMS (rTMS) where rapid series (‘trains’) of pulses are delivered (≥1 Hz). TMS can be combined with electroencephalography (EEG) or electromyography (EMG) as a readout of the cortical changes evoked by the stimulation (Rothwell et al., 1987). The concurrent EEG or EMG records the TMS-induced cortical activation in real time (Rothwell et al., 1987, Komssi and Kähkönen, 2006). Within this framework several biomarkers have been developed in order to measure the levels of cortical excitation and inhibition, and their ratio (E:I ratio). These measures include the motor threshold (MT), cortical silent period (CSP), short interval intracortical inhibition (SICI), intracortical facilitation (ICF), and long interval intracortical inhibition (LICI) (Rossini et al., 2015). Although there is not robust evidence yet on the role of these measures as biomarkers in epilepsy, they are likely to predict response to treatment and pathophysiology in people with epilepsy (Bauer et al., 2018, Silvennoinen et al., 2020, Tsuboyama et al., 2020). When coupled with EEG, TMS can evoke potentials (TMS-Evoked Potentials, TEPs) that are a measure of the cortical activation and also represent a potential biomarker for diagnosis and treatment in epilepsy (Amassian et al., 1992, Virtanen et al., 1999, Kimiskidis et al., 2014, Kimiskidis, 2016, ter Braack et al., 2016, Tremblay et al., 2019). There are previous reports of people with and without epilepsy experiencing seizure activity during or after sp/ppTMS sessions (Hömberg and Netz, 1989, Fauth et al., 1992, Classen et al., 1995, Michelucci et al., 1996, Haupts et al., 2004, Tharayil et al., 2005, Kratz et al., 2011). Although there is no evidence that sp/ppTMS may induce permanent neuronal changes, there are safety concerns around stimulation of the brain and its effects in individuals with neurological or psychiatric conditions (Wolpe, 2002, Illes, 2006). Multiple sets of TMS safety guidelines have been issued over time to minimise the risk of TMS-induced adverse events (Wassermann, 1998, Rossi et al., 2009, Rossi et al., 2020). Previous studies have determined a low risk of seizure induction by sp/ppTMS (Schrader et al., 2004, Lerner et al., 2019, Chou et al., 2020), and suggested that ‘high cortical excitability states’ may be associated with generation of TMS-induced epileptiform discharges (Kimiskidis et al., 2015). However, the exact mechanisms by which seizures may be induced during or after TMS are unknown, and it is not possible to quantify specific risks and effects based on the available evidence. These uncertainties may affect study approval and subject recruitment to TMS research (Heinrichs, 2012). The aim of this study was to assess the effects of sp/ppTMS on people with and without epilepsy. This was achieved through analysis of the clinical course of seizures before and after TMS exposure.

## 2 Methods

The study was approved by the local ethical review board (National Research Ethics Service Committee London–Camden and Islington) for TMS on PWE (REC reference EpiPGX, 11/LO/2016). For controls, the study was approved by the ethical review board (London South East Research Ethics Committee) to conduct all the experiments on healthy controls or people with genetic neurological conditions without epilepsy (Cortical Excitability, REC reference 15/LO/1642). All experiments were conducted at the Chalfont Centre for Epilepsy, Buckinghamshire, UK.

### 2.1 Participants

We included in the study PWE and controls who underwent TMS consecutively from 1.04.2019 to 15.03.2020, after providing written informed consent. Exclusion criteria included: participants who had medical records of intracranial metallic implants, drug released dispensers, metallic tattoos, cardiac pacemakers, and pregnant women. An approved adapted information sheet and consent form were used for individuals with mild learning disabilities. For participants lacking capacity, parental or legal guardian assent was provided.

### 2.2 Recording setups and montages

**Fig. 1:**
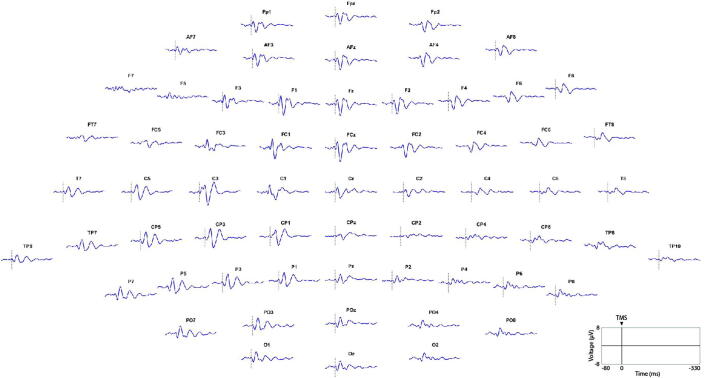
63-sensor EEG montage with topographical distribution of the average responses for each EEG channel between –80 ms and +330 ms. Stimulation was applied to the motor left area (BA4 Left). Original image generated using MATLAB 2017a script incorporating SPM12 and EEGLAB v.13 (Delorme and Makeig, 2004, Ashburner et al., 2014).

High-density EEG (HD-EEG) recording setup consisted of an actiCHamp 64-channel amplifier (Brain Products, GmbH, Germany) and TMS-compatible actiCAP slim active electrodes. Electrodes were placed in a 10–20 configuration [Fig. 1]. For some subjects, vertical electrooculography (EOG) was recorded by using two extra sensors. All equipment ensured TMS-artefact-free EEG recordings starting around 5 ms after a single TMS pulse (Rosanova et al., 2009). EEG recording procedure was adapted from previous research (Casali et al., 2010). EMG recordings were acquired from the right first dorsal interosseus (FDI) muscle using 15 by 20-millimetre bipolar surface electrodes (Unimed Electrode Supplies Ltd., Surrey, UK) placed in a belly tendon montage three centimetres apart. EMG signals were amplified (0.05–0.1 ÂµV), band pass filtered (high pass 5 Hz; low pass 2000 Hz; notch 50 Hz) and digitised (sampling rate 5 kHz) with a Signal interface system (Cambridge Electronic Design Co. Ltd., United Kingdom). At the start of each experiment, the motor ‘hotspot’ for the FDI muscle was located over the contralateral primary motor cortex.

Subjects were asked to wear a pair of noise-cancelling headphones (Shure SE215-CL-E Sound Isolating) that played a white masking noise. The masking noise contained the same spectral profile of the TMS profile click to avoid TEP signal contamination caused by auditory responses to clicks produced by the TMS coil. The usage of white and masking sounds allowed the data capture of specific time-varying frequency components of the TMS coil click. If a participant reported hearing a click during the session, the volume of the masking sound was increased to a level that was still comfortable for the subject and the real TMS click was not perceived (Rosanova et al., 2009, Rosanova et al., 2018). Masking noise volume was kept under 90 dB for all participants and only used in sessions with TMS.

A figure of eight coil with 70-millimetre diameter driven by a monophasic stimulator Magstim 2002 (Whitland, UK) was used for all experiments. For TMS-EEG, stimulation intensity was kept between 33 and 100% maximum stimulator output (MSO) as guided by the resting motor threshold (RMT) that ranged between 37 and > 100% MSO. Single pulses were delivered with an interstimulus interval (ISI) of 1.9–3.3 s. Pulse delivery was controlled by the Signal software (Cambridge Electronic Design Co. Ltd., United Kingdom). At least 100 pulses were delivered during each single pulse assessment. For paired-pulse TMS-EMG, two Magstim 2002 stimulators were connected by a Bistim2 unit (Magstim, Whitland, UK). Test stimulus intensity was set to produce motor evoked potentials (MEP) of approximately 1 millivolt. Conditioning stimulus was set at 70% RMT. For SICI, ISIs of 2 and 3 ms were used. For ICF, the ISI was 15 ms. A total of 100–120 pulses were delivered during each paired pulse assessment.

### 2.3 TMS-EEG data acquisition

Participants were asked to be seated comfortably in a TMS-compatible chair for the entirety of the TMS study. HD-EEG and EMG sensors were applied. At the start of the experiment, RMT was determined, defined as the TMS intensity required to elicit MEPs of >50 µV in at least five out of ten consecutive trials delivered every 5 s or more (<0.2 Hz) (Rossini et al., 2015).

**Fig. 2:**
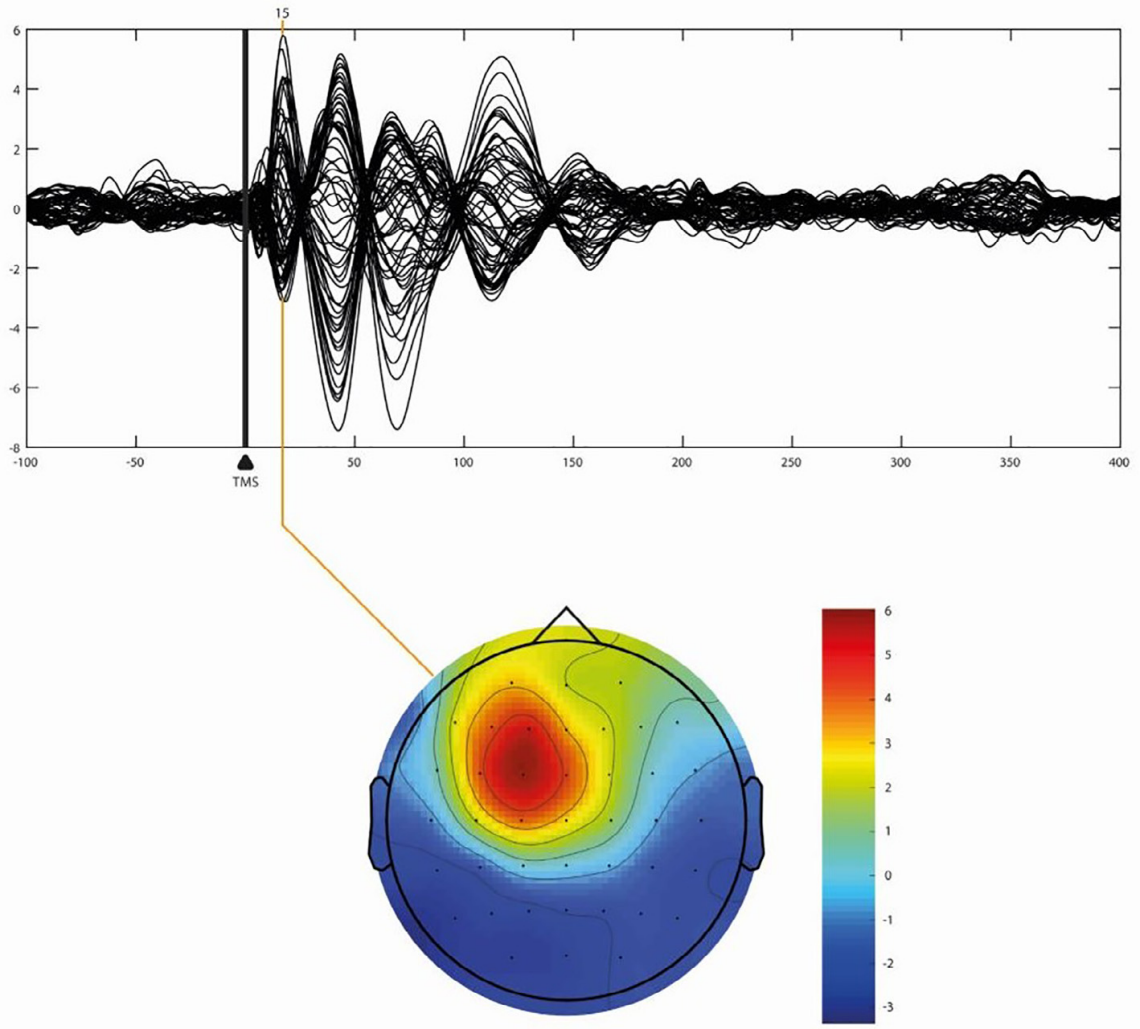
Butterfly plot of a representative subject stimulated in the left motor area (BA4 Left) capturing the average response for each EEG channel between –100 ms and + 400 ms displayed in average reference (Delorme and Makeig, 2004, Ashburner et al., 2014).

Cortical areas were targeted by using the individual structural MRI (available for 32 PWE, 2 AHC, 8 controls) or a template MRI from Brainsight software, i.e. Brainbox, (3 PWE, 10 controls). The Brainsight software implemented a 3D infrared Tracking Position Sensor Unit to map the position of the TMS coil on the subject’s head within the reference space of the individual structural MRI to facilitate targeting. Target areas included: premotor (Brodman’s area, BA6), motor (BA4), parietal areas (BA7), occipital area (BA19), bilaterally. Selected areas were in keeping with previous research (Rosanova et al., 2009, Casarotto et al., 2010). The TMS coil was placed tangentially to the subject’s scalp with the coil centre overlaying the target site, so as to direct the TMS-induced current perpendicularly to the stimulated gyrus. *In vivo* online visualisation of the TEPs further guided the coil orientation to avoid muscle artefacts and maintain optimal signal-to-noise ratio rate [Fig. 2].

A single TMS with HD-EEG session lasted approximately 2–3 h, collecting an average of 150–200 TMS trials and two resting-state EEG sessions per subject. Paired pulse TMS-EMG was performed in a total of three patients to measure short-interval intracortical inhibition (SICI) and intracortical facilitation (ICF) was also obtained in one patient. We used established stimulation parameters (Kujirai et al., 1993, Rossini et al., 2015).

Due to the retrospective design of the study, resting EEG sessions before and after TMS were available for a minority of individuals. Resting EEG was recorded for at least 4 min, before and after TMS, and including ‘eyes open’ and ‘eyes closed’ conditions. As the TMS sessions were long and often tiring for the participants, the resting EEG could not be recorded for a prolonged time; participants were awake during the resting EEG recording, EEG signals were band-pass filtered between 0.1 and 500 Hz and sampled at 5000 Hz with 32-bit resolution. Electrode impedance was kept below 10 kV. [For details of the EEG analysis see Appendix, Methods A1 and Appendix Table A1.].

### 2.4 Clinical data acquisition

The study design employed a retrospective acquisition of clinical data consisting of follow-up telephone interviews and analysis of electronic health records for further clinical history (e.g., seizure frequency, treatment change, comorbidity). The aim of the interview was to evaluate acute and chronic effects of TMS on seizure frequency, and adverse events. Participants were asked for the number of seizures in the four weeks before and after their TMS session from their seizure diaries, and whether they experienced any noticeable changes after the study that may have impacted their overall wellbeing. Change in seizure frequency was defined as an increase or decrease of 25% or higher (Birbeck et al., 2002). Parents and caregivers were asked to provide commentary on behalf of participants with intellectual disability.

### 2.5 Statistical analysis

Clinical information was inputted and coded to identify whether subjects with epilepsy had any of the following changes comparing the four weeks before with the four weeks after TMS assessment: (i) Any treatment change, (ii) ASM introduction, (iii) ASM withdrawal, (iv) ASM dosage increase, (v) ASM dosage decrease, (vi) change in other medication with effect on the central nervous system (CNS) (non-ASM), (vii) Any change in seizure frequency after TMS, (viii) Increase in seizure frequency after TMS, and (ix) Number of seizures four weeks before and after TMS. Data were assessed using STATA 15 (Statacorp LLC, College Station, Texas, USA). Fisher’s Exact tests and Chi Square tests, as appropriate, were used in the analyses to determine association between treatment changes with change in seizure frequency after TMS. Seizure frequencies four weeks before and after the study were captured as a continuous variable and were compared using a Wilcoxon signed rank test. Two-tailed *P*-values lower than 0.05 were considered statistically significant.

A *post hoc* power analysis estimated a power of 0.84 to detect a seizure increase or decrease above 50% given the sample size of 35 PWE, with a significance level of 0.05 (α) (two-sided *t*-test).

## 3 Results

### 3.1 Subject demographics

We studied 35 PWE (14 males, 21 females, mean = 32.97 years, SD = 10.81 years, range = 19–60 years) [Table 1]. Age of epilepsy onset ranged from 2 weeks to 40 years (mean = 7.38 years, SD = 9.55 years) [Table 1].

**Table 1:** Demographic, clinical and treatment data of epilepsy cohort.

Patient	Age	Sex	Diagnosis	Comorbidities	Medications 4 weeks before TMS	Medications 4 weeks after TMS
1	22	M	Dravet Syndrome with *SCN1A* mutation, DRE	Intellectual disability; short stature; ECG dynamic abnormalities	Bromide on gradual withdrawal; Valproate; Levetiracetam; Buccal midazolam	Bromide re-introduced due to seizure worsening. Other medications unchanged.
2	19	M	Multifocal epilepsy, DRE	Severe intellectual disability; band heterotopias; colostomy; PEG feeding tube *in situ*; central apnoea with home oxygen; urinary retention	Phenobarbital; Levetiracetam; Buccal midazolam	Medications unchanged.
3	33	F	Alternating hemiplegia; seizures	Bilateral lower limb spasticity; headache	Phenytoin; Topiramate; Baclofen; Cinnarizine	Medications unchanged.
4	22	M	Dravet syndrome with *SCN1A* mutation, DRE	Severe intellectual disability; autistic spectrum disorder; behavioural difficulties; dyspraxia; dysautonomia; sleep disturbance; lax ligaments	Clobazam; Sodium valproate; Stiripentol; Melatonin; VNS	Medication unchanged.
5	20	M	Dravet syndrome with *SCN1A* mutation, DRE	Intellectual disability; history of adverse reaction to phenytoin	Brivaracetam; Sodium valproate; Clobazam; Midazolam.	Medications unchanged.
6	30	M	Dravet syndrome with *SCN1A* mutation, DRE	Intellectual and behavioural difficulties; nocturnal episodes of arousal and vocalisation; spastic diplegia	Zonisamide; Sodium valproate; Clobazam, Levetiracetam; VNS *in situ.*	Medications unchanged.
7	30	M	Dravet Syndrome of *SCN1A* mutation, DRE	Mild learning disability	Stiripentol; Zonisamide; Sodium valproate; Buccal midazolam; Clobazam; VNS *in situ*	Medications unchanged.
8	38	F	Dravet Syndrome with *SCN1A* mutation, DRE	Severe intellectual disability; previous episodes of phenytoin toxicity	Stiripentol; Levetiracetam; Epilim chrono	Epidiolex was introduced. Other medications unchanged.
9	36	F	Dravet Syndrome with *SCN1A* mutation, DRE	Bilateral lower limb swelling	Zonisamide; Sodium valproate	Epidiolex was introduced. Other medications unchanged.
10	22	F	Dravet syndrome with *SCN1A* mutation, DRE	Intellectual and behavioural difficulties; scoliosis; eczema; mild thrombocytosis; hypovitaminosis D	Sodium valproate; Stiripentol	Epidiolex was introduced. Other medications unchanged.
11	51	M	Dravet Syndrome with *SCN1A* mutation, DRE	Intellectual disability; headache	Levetiracetam; Clonazepam; Sodium valproate; Amitriptyline	Epidiolex was introduced. Other medications unchanged.
12	25	M	Alternating hemiplegia of childhood with epilepsy	Orofacial dyspraxia; intellectual disability; recurrent right knee dislocation; headaches	Topiramate; Pizotifen; Flunarizine	Medications unchanged.
13	37	F	Dravet Syndrome with *SCN1A* mutation, DRE	Dysphagia requiring PEG insertion	Valproate liquid; Levetiracetam liquid; Oxcarbazepine liquid	Epidiolex was introduced. Other medications unchanged.
14	36	F	Alternating hemiplegia of childhood with epilepsy	Palpitations; microvascular angina; previous psychosis	Flunarizine; Topiramate; Pizotifen; Promethazine; Baclofen; Clonazepam; Zonisamide; Ketogenic diet.	Medications unchanged.
15	47	F	Idiopathic generalised epilepsy, DRE	Bipolar disorder; memory difficulties; longstanding backpain; bilateral hearing loss; asthma	Aripiprazole; Carbamazepine; Lamotrigine; Omeprazole; Clobazam	Medications unchanged.
16	29	M	Epilepsy in remission, previous history of Landau-Kleffner syndrome	Intellectual disability	Oxcarbazepine; Levetiracetam; Clobazam	Medications unchanged.
17	23	F	Idiopathic generalised epilepsy, family history of epilepsy	Mood disturbance with anxiety	Zonisamide; Levetiracetam; Duloxetine; Diazepam.	Zonisamide increased. Propranolol started. Diazepam was stopped. Other medications unchanged.
18	31	F	Rasmussen's encephalitis, DRE	Right homonymous visual field defect; non-epileptic attack disorder	Lacosamide; Levetiracetam; Azathioprine; Clobazam; Colecalciferol; Ferrous fumarate; Omeprazole; Phenytoin sodium; Tramadol	Medications unchanged.
19	47	F	Focal epilepsy associated with *DEPDC5* mutation, DRE	Mild cognitive impairment; migraine; constipation	Carbamazepine on gradual withdrawal; Lamotrigine; Topiramate; Propranolol.	Carbamazepine withdrawn. Topiramate increased. Lamotrigine reduced. Other medications unchanged.
20	23	M	Focal epilepsy, DRE	Static intellectual disability, autistic spectrum disorder, gluten intolerance and eosinophilic colitis	Carbamazepine; Lamotrigine; Propranolol; Cannabidiol; Ketogenic diet	Medications unchanged.
21	26	F	Focal epilepsy; Tuberous sclerosis	Psychosis; intellectual disability; autism Spectrum Disorder; anxiety	Aripipirazole; Vigabatrin; Lacosamide; Procyclidine	Medications unchanged.
22	22	F	Idiopathic generalised epilepsy; DRE; family history of epilepsy	Intellectual disability	Clonazepam; Perampanel; Zonisamide; Ethosuximide on gradual withdrawal; Midazolam	Zonisamide on gradual withdrawal. Ethosuximide stopped. Brivaracetam was introduced. Other medications unchanged.
23	25	F	Unclassified epilepsy, DRE	Migraine; polycystic ovarian syndrome; depression and anxiety	Levetiracetam; Lamotrigine; Zonisamide; Clobazam; Buccal midazolam	Levetiracetam stopped. Zonisamide increased. Other medications unchanged.
24	33	M	Focal epilepsy, DRE		Zonisamide; Valproate; Brivaracetam; Clobazam	Medications unchanged.
25	34	F	Focal epilepsy, DRE		Lamotrigine; Perampanel; Epilim Chrono; Zonisamide; Clobazam	Perampanel increased and Zonisamide reduced. Other medications unchanged.
26	30	F	Focal epilepsy, DRE	Hypovitaminosis D	Lamotrigine	Medications unchanged.
27	44	F	Focal epilepsy; Focal cortical dysplasia	Endometriosis	Levetiracetam; Adcal	Medications unchanged.
28	60	F	Focal epilepsy	Coeliac disease	Levetiracetam; Clobazam	Medications unchanged.
29	33	M	Idiopathic generalised epilepsy	Mood lability	Sodium valproate; Zonisamide; Citalopram	Medications unchanged.
30	40	F	Rasmussen's encephalitis, DRE	Non-epileptic attack disorder	Sodium valproate; Pregabalin; Phenytoin; Oxcarbazepine; Levetiracetam; Clobazam; Ranitidine; Ondansetron	Medications unchanged.
31	29	F	Dravet syndrome with *SCN1A* mutation, DRE	Learning disability	Perampanel; Sodium valproate; Carbamazepine; Clobazam	Medications unchanged.
32	26	F	Epilepsy, classification unclear, DRE	Cognitive difficulties; mild renal impairment	Perampanel; Lamotrigine; Zonisamide	Perampanel increased. Other medications unchanged.
33	25	M	Focal epilepsy, DRE	Left parietal, occipital, temporal hypoxic ischaemic injury	Oxcarbazepine; Sulthiame; Cannabidiol; Phenobarbitone; Lorazepam	Phenobarbitone increased. Other medications unchanged.
34	46	F	Focal epilepsy, DRE	Left frontal cortical thickening	Lacosamide; Oxcarbazepine; Pregabalin	Lacosamide on gradual withdrawal.
35	60	M	Idiopathic generalised epilepsy	Non-epileptic attack disorder	Sodium valproate; Lamotrigine	Medications unchanged.

Abbreviations: DRE = Drug resistant epilepsy; VNS = vagus nerve stimulator.

The control cohort comprised 18 subjects [Appendix, Tables A2 and A3], including 16 healthy participants (9 males, 7 females, mean = 36.13 years, range = 28–60 years, SD = 10.31) and two additional subjects (2 females, mean = 32.5 years, range = 24–41 years, SD = 12 years) with alternating hemiplegia of childhood (AHC), with no clinical diagnosis of epilepsy or seizure history (Heinzen et al., 2012).

**Fig. 3:**
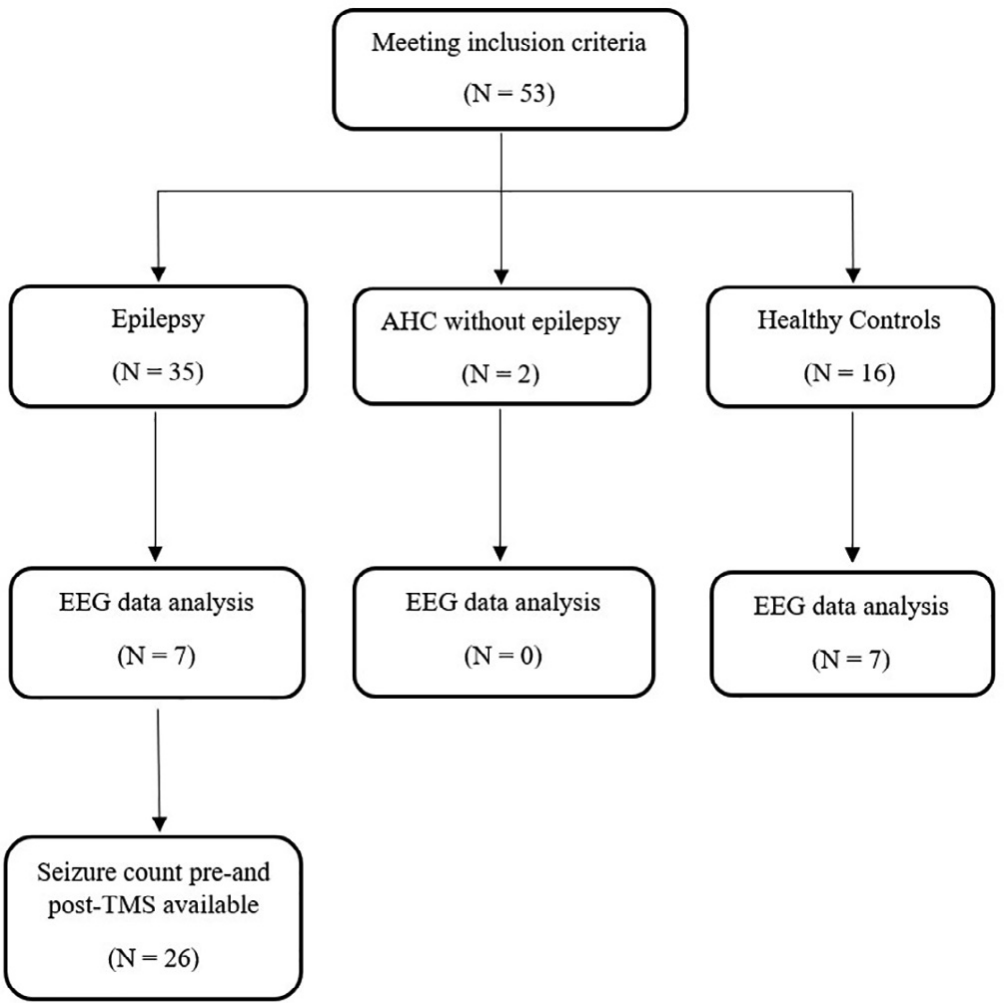
Subject cohort allocation.

Twenty-nine of the 35 subjects with epilepsy participated in the clinical follow-up interview. Four subjects were excluded due to unavailable data on seizure frequency within the four weeks before and after TMS. We included in the analysis 26/35 (74%) PWE. The two controls with AHC with no history of epilepsy were also contacted to provide retrospective commentary on any changes in frequency of hemiplegic episodes and if spontaneous seizure activity had occurred after the study [Fig. 3].

### 3.2 Clinical course

Twenty-six PWE with seizure diaries provided accurate (i.e. daily) count of seizure frequencies during four weeks before and after the TMS session. Five PWE did not have an accurate daily recording of their seizures but were all able to provide a weekly figure for their seizure frequency before and after TMS. Four participants in the epilepsy cohort were seizure-free having either controlled epilepsy or epilepsy in remission (Sillanpää et al., 2017). Subjects classified as seizure-free had no seizures for at least 12 months before the study. All four subjects remained seizure-free after TMS and were included in the seizure frequency analysis. Healthy controls did not experience any seizures during or after the TMS study. The two subjects with AHC with no epilepsy reported no seizure activity and no changes in hemiplegic episode frequency in the four weeks after TMS.

**Fig. 4:**
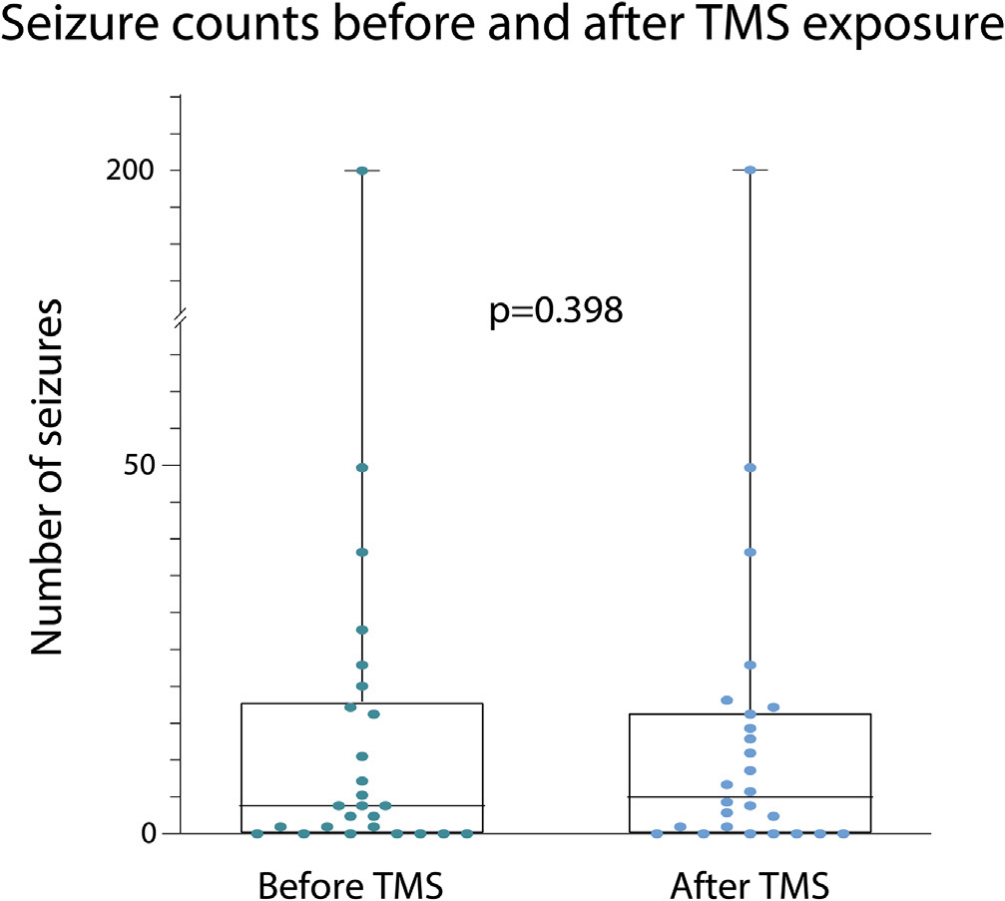
Graph of the number of seizures before and after TMS for each PWE who kept a seizure diary and recorded the frequencies from four weeks leading up to the study and four weeks after (n = 26). Each data point represents a different subject. The y axis is a non-linear scale (the values between 70 and 180 were omitted as no seizure count resulted in that window). Statistical comparison was performed with the Wilcoxon signed rank test.

There was no significant difference in seizure frequency before and after TMS across the whole cohort of both PWE and AHC subjects with data available (*p* = 0.398) [Fig. 4], also after excluding the four individuals who were seizure-free (*p* = 0.400). At the individual level, change in seizure frequency in the four weeks after TMS was reported in 13 PWE (37%), including increase (n = 8) or decrease (n = 5) [details provided in Appendix Table A4]. Of the 30 PWE and AHC subjects with accurate diaries including daily seizure frequency, 9/30 (30%) had seizures which occurred within the 24 h after the TMS session but only one of them had seizure increase in the following four weeks. Treatment changes, consisting of ASM introduction, withdrawal, dosage increases, dosage decreases, and medications not classified as ASMs but with effect on CNS, were included in the analysis. In total, 14 PWE (40%) had medication changes in the four weeks before TMS, due to uncontrolled seizures in the majority (n = 13) or to side effects (n = 1). We observed that 20 PWE (57%) had ASM changes during the eight weeks preceding the study, with no significant difference when compared to the number of PWE who required ASM changes during the study period (*p* = 0.635). PWE who had seizure changes (either increase or decrease) after TMS more frequently had introduction of a new ASM before TMS [Table 1], compared to PWE with stable seizure frequency (7 vs 1, *p* = 0.016), as would be expected. There were no changes in other medications with effect on CNS (other than ASMs) in the four weeks before or after TMS.

### 3.3 Adverse events

During clinical interviews, participants were encouraged to give any feedback on their overall wellbeing after the study. While most reported no adverse events, four PWE (11%) experienced discomfort (n = 1), mild headaches (n = 1), or body heaviness (n = 2) during or immediately after their session. These responses are in line with possible minor side effects of TMS administration reported already (Rossi et al., 2009). No adverse events were reported in the two subjects with AHC without epilepsy.

[Results of the resting EEG analysis are presented in Appendix Results A2.].

## 4 Discussion

TMS is a promising non-invasive research tool that can provide information on cortical excitability and connectivity in both clinical and healthy populations. Recent guidelines estimate a risk lower than < 0.03% of seizure induction during TMS (Wassermann, 1998, Schrader et al., 2004, Lerner et al., 2019, Chou et al., 2020); however, there are no systematic data on the effects of sp/ppTMS at the electrophysiological and clinical level. Most previous reports of seizure incidence in sp/ppTMS were anecdotal, presenting single events with inconclusive results and research parameters that may not meet current TMS research guidelines (Hömberg and Netz, 1989, Fauth et al., 1992, Classen et al., 1995, Michelucci et al., 1996, Haupts et al., 2004, Tharayil et al., 2005). Additionally, study design heterogeneity, small sample sizes and diversity of clinical profiles are all potential confounding factors that may influence risk estimation (Tremblay et al., 2019). Adverse events may derive from external factors aside from TMS (e.g., sleep deprivation, participation anxiety, convulsive syncope, medication usage, or natural fluctuations of existing neurological conditions) (Wolpe, 2002, Kratz et al., 2011, Bauer et al., 2014). Therefore, events that may resemble seizure activity require further *post hoc* analysis to determine semiology prior to classification as an epileptic seizure.

Meta-analyses and guidelines that have attempted to quantify the risk of sp/ppTMS-related seizure induction have estimated a minimal risk based on the existing evidence of sp/ppTMS provoking seizures in PWE (Schrader et al., 2004, Rossi et al., 2009, Rossi et al., 2020, Lerner et al., 2019, Chou et al., 2020). Aligning with these findings, our clinical data analysis revealed no significant statistical difference in seizure frequency at an individual level in PWE after TMS assessment, providing additional evidence that sp/ppTMS are safe in PWE.

The majority of PWE had a diagnosis of DRE [Table 1]. Treatment change due to uncontrolled seizures were ongoing in this population, and thus may represent a confounding factor in our TMS cohort. ASM adjustment was required in 17 PWE before or after TMS, and in 12 PWE ongoing seizure increase was reported after TMS including four PWE with increase above 50%; this is likely due to either treatment change being ineffective or to the too short time to determine the effect of the changes. We found no significant difference between the prevalence of medication changes required during our study period and the one observed over the eight weeks preceding our study, suggesting that our cohort was representative of a drug-resistant population with fluctuations in seizure control often requiring medication changes. In the subgroup with available seizure count, there was no significant difference after TMS. On this basis, we ascribe seizure frequency changes after TMS to the habitual fluctuations in seizure frequency in DRE, rather than to direct impact of TMS. TMS did not induce seizure activity in the control cohort during or after the experiment. It is worth highlighting that there were also four individuals in the epilepsy cohort who were seizure free for at least 12 months at the time of participation. These four PWE remained seizure free after the study over the period of observation and had no changes in treatment in the four weeks after the TMS experiment. This suggests that sp/ppTMS is not only unlikely to provoke seizures in individuals without epilepsy, but also not likely to alter cortical activity and induce seizures in those with controlled epilepsies.

Resting-state HD-EEG data showed no significant electrophysiological changes post-TMS. All cohorts had similar results, with stable background activity with little or no activation of epileptiform activity in PWE. The combination of clinical and EEG data in our study provides additional evidence that sp/ppTMS protocols are safe in populations with and without epilepsy. Our findings are consistent with a comparable study performed by Tassinari and colleagues, who provided initial proof-of-concept that spTMS neither acutely nor chronically impacts seizure frequency (Tassinari et al., 1990). In their assessment of 58 PWE, only one subject experienced seizure activity during TMS, with *post-hoc* EEG analysis concluding that seizure activity at the time of TMS was attributable to the habitual seizure frequency of the patient rather than TMS itself (Tassinari et al., 1990). We note the main methodological differences between our study and Tassinari’s include the use of HD-EEG vs 16-channel EEG recording, and high (>100) vs low (<30) number of stimuli in each TMS session (Tassinari et al., 1990). An additional novel finding in our study is the safety of TMS in vulnerable PWE, such as patients with Dravet Syndrome and other syndromes with intellectual disability and severe epilepsy.

Previous metanalyses and reviews have attempted to quantify the risk of whether TMS has a risk of eliciting seizures and found that a risk of inducing seizures of 0–2.8% for spTMS and 0–3.6% for ppTMS (Schrader et al., 2004). Likewise, Lerner and colleagues showed an average risk of spTMS and ppTMS inducing seizures of 0.12/1000 sessions, based upon a survey of TMS studies conducted between 2012 and 2016 (Lerner et al., 2019). Our finding that sp/ppTMS does not induce seizure activity during testing may be due to the experimental stimulation frequencies being below 0.53 Hz. This should be a consideration when deciding upon TMS testing specifications. Further research is warranted to investigate whether stimulation above 0.53 Hz may increase the risk of seizure provocation, and whether there might be a differential impact of spTMS compared to spTMS if delivered at frequencies below 0.5 Hz.

### 4.1 Study limitations

One study limitation is the small and heterogenous size of our clinical cohort. Our epilepsy cohort included different epilepsies, seizure types and comorbidities [Table 1] which may represent potential confounding factors. Data quality was noticeably lower in recordings that were performed after TMS compared to baseline recordings across all cohorts. Post-TMS resting EEG recordings were visibly more artefactual and had higher sensor impedances [example visualisations are in Appendix Fig. A2]. Due to artefactual data, only EEG from 14 subjects could be used for analysis. Lower data quality could be ascribed to study length, as a single TMS experimental session typically ranged 2–3 h and subjects may have been increasingly restless or drowsy towards the end of the study. Sensors may need to be checked and optimised before restarting a resting EEG session recording at the end of TMS experiments. The duration of the resting EEG recordings may have been relatively short for capturing low-density epileptiform discharges.

Due to the retrospective study design, there was incomplete data from seizure diaries when monitoring changes that occurred in the four weeks leading up to the study and four weeks after. Only 26 respondents from the epilepsy cohort were able to provide accurate seizure counts at clinical interviews. The remaining nine subjects did not report accurate seizure counts due to either the lack of a proper seizure diary or incomplete diary entries. We also note that our cohort included individuals with Dravet Syndrome or other type of epilepsy syndromes with intellectual disability; although most carers were able to provide accurate data on their seizure frequency, there is a limitation in their ability to report adverse events.

## 5 Conclusions

We provide a comprehensive analysis of the impact of TMS on epilepsy activity by examining spTMS and ppTMS research paradigms and using clinical assessment up to four weeks after TMS. Our findings provide additional evidence on the safety of spTMS and ppTMS protocols in people with and without epilepsy.

## Author contributions

S Pang and S D’Ambrosio contributed to drafting of the manuscript, data analysis, and creation of the figures. G Battaglia, D Jiménez-Jiménez, M Perulli, K Silvennoinen, S Zagaglia, contributed to data collection and analysis. SM Sisodiya contributed to critical revision of the manuscript. S Balestrini contributed to the study design and critical revision of the manuscript.

## Declaration of competing interest

None.

## Ethical publication statement

We confirm that we have read the Journal’s position on issues involved in ethical publication and affirm that this report is consistent with those guidelines.

## Funding

This work was supported by a grant from the 10.13039/100015873National Brain Appeal (NBA).

UCB provided financial support for SD. UCB had no editorial control and no input or decision over the selection of authors or topics discussed.

SD, SB and SMS are supported by the Epilepsy Society. SB was supported by the Muir Maxwell Trust. Part of this work was undertaken at University College London Hospitals, which received a proportion of funding from the NIHR Biomedical Research Centres funding scheme. KS is supported by a Wellcome Trust Strategic Award (WT104033AIA).
