# Supplementary material for: The impact of Transcranial Magnetic Stimulation (TMS) on seizure course in people with and without epilepsy
Source: Clin Neurophysiol Pract. 2022 Jun 13;7:174–82. doi: 10.1016/j.cnp.2022.05.005 (PMC9254260; doi:10.1016/j.cnp.2022.05.005)
Supplement: Supplementary data 1 [file mmc1.docx]

**Appendix**

**Methods A1. EEG analysis.**

HD-EEG data analysis was performed using ASA PRO 8 (ANTneuro, Hengelo, The Netherlands) and EDF Browser (GNU General Public License v3). EEG recordings for epilepsy and control cohorts were visually inspected by two neurophysiologists. The EEG was visualised in both bipolar and referential (recorded to a common reference) montages according to the International 10-20 System. When the common average reference was used, channels showing large artefacts were removed from the display. A modified version of the Standardised Computer-based Organized Reporting of EEG (SCORE) was used to analyse the resting-state EEG recordings (Beniczky et al., 2017) [See Appendix Table A1]. McNemar’s test was used to compare the presence of interictal EEG abnormalities before and after TMS.

**Table A1.** EEG features evaluated for epilepsy and non-epilepsy control cohorts.

| Cohort | Neurophysiological Features | Scoring |
| --- | --- | --- |
| Epilepsy | Background activity | Categorical:  ‘Normal posterior dominant alpha band’; ‘Abnormal slow alpha activity’;  ‘Background activity not assessable’ |
|  | Frequency of background activity | Categorical: Alpha; Theta; Delta; Beta |
|  | Epileptiform interictal activity | Binary:  Yes/no |
|  | Spike |  |
|  | Spike and slow wave |  |
|  | Polyspikes |  |
|  | Polyspikes and slow wave |  |
|  | Sharp wave |  |
|  | Rapid spikes |  |
| Non-epilepsy controls | Background activity | Categorical:  Normal (posterior dominant rhythm alpha band);  Abnormal (slow) |
|  | Frequency of background activity | Categorical: Alpha; Theta; Delta; Beta |
|  | EEG slowing | Categorical: None; Non-rhythmic; Rhythmic    Categorical: Anterior; Posterior; Multifocal |
|  | Mode of appearance | Categorical: Random; Periodic; Variable |
|  | Presence of abnormal interictal rhythmic activity | Binary: Yes/No |

**Table A2.** Demographic data of the healthy controls.

| Subject | Sex | Handedness | Age (years) |
| --- | --- | --- | --- |
| 1 | F | R | 35 |
| 2 | M | R | 34 |
| 3 | F | L | 30 |
| 4 | F | R | 31 |
| 5 | M | R | 29 |
| 6 | F | R | 31 |
| 7 | F | R | 30 |
| 8 | F | R | 28 |
| 9 | F | R | 29 |
| 10 | F | R | 33 |
| 11 | F | R | 37 |
| 12 | M | R | 46 |
| 13 | M | R | 62 |
| 14 | M | R | 58 |
| 15 | M | R | 35 |
| 16 | M | R | 30 |

**Table A3.** Clinical information of the control subjects with Alternating Hemiplegia of Childhood (AHC) due to mutation in *ATP1A3* with no diagnosis of epilepsy.

| Subject | Age | Sex | Diagnosis | Comorbidities | Medications 4 weeks before TMS | Medications 4 weeks after TMS |
| --- | --- | --- | --- | --- | --- | --- |
| 1 | 24 | F | Alternating hemiplegia of childhood due to mutation in *ATP1A3* | (1) Intellectual disability (2) Hay fever | Buccal midazolam as required | No change in medication. |
| 2 | 41 | F | Alternating hemiplegia of childhood due to mutation in *ATP1A3* | (1) Verbal outbursts | Baclofen | No change in medication |

**Table A4.** People with epilepsy who had change in seizure frequency in the 4-week interval after TMS. The seizure count percentage was calculated by subtracting the number of seizures between counts after TMS and those before TMS. Any change +/-25% or higher was determined as a change of seizures.

| **Subject Number** | **Number of Seizures 4 weeks before TMS** | **Number of Seizures 4 weeks after TMS** | **Percent change** |
| --- | --- | --- | --- |
| 4 | 11 | 13.5 | 23% |
| 5 | 195 | 203 | 4% |
| 6 | 4 | 4.5 | 13% |
| 7 | 4 | 7 | 75%* |
| 8 | 29 | 19 | -34%* |
| 9 | 7.5 | 11.5 | 53%* |
| 10 | 21 | 18 | -14% |
| 11 | 2.5 | 3 | 20% |
| 13 | 18 | 17 | -6% |
| 18 | 17 | 15 | -12% |
| 19 | 1 | 9 | 800%* |
| 31 | 4 | 6 | 50%* |
| 32 | 5.5 | 4 | -27%* |

*= Change in seizure frequency was defined as an increase of decrease of 25% or higher.

**Results A2. EEG analysis.**

Resting EEG recordings were available for 7/35 (20%) PWE, and none of them were seizure-free at time of study. We reviewed an average of 4.38 minutes (SD ±0.29) of resting EEG before TMS and an average of 4.31 minutes (SD ±0.48) after TMS. Of the seven subjects, four had normal alpha background activity and three had abnormal alpha background activity marked by slowed posterior alpha waves. Six participants had no changes in background activity or epileptiform discharges after TMS. One subject had occasional epileptiform discharges (i.e., polyspike and slow waves) observed in the EEG post-TMS, whilst there was no epileptiform activity recorded in the EEG pre-TMS [Example visualisations can be found below in Appendix Figure A1]. Overall, there was no significant difference in EEG features before and after TMS (*p*=1.000). Further details can be found in Appendix Table A5.

Seven resting-EEG recordings were available for healthy controls (7/16, 44%) with an average duration of 9 minutes (SD ±2) before TMS and 9 minutes (SD ±2) after TMS. There was no slowing of the EEG rhythms nor epileptiform discharges appearing in any of the post-TMS recordings. Healthy controls had no seizure history and did not experience seizure activity during or after TMS.

**Table A5.** EEG findings in people with epilepsy before and after TMS. A total of 63.09 minutes of EEG recordings were reviewed for this cohort. The EEG assessment was based on the SCORE EEG reporting (Beniczky et al., 2017), see Methods for further reference.


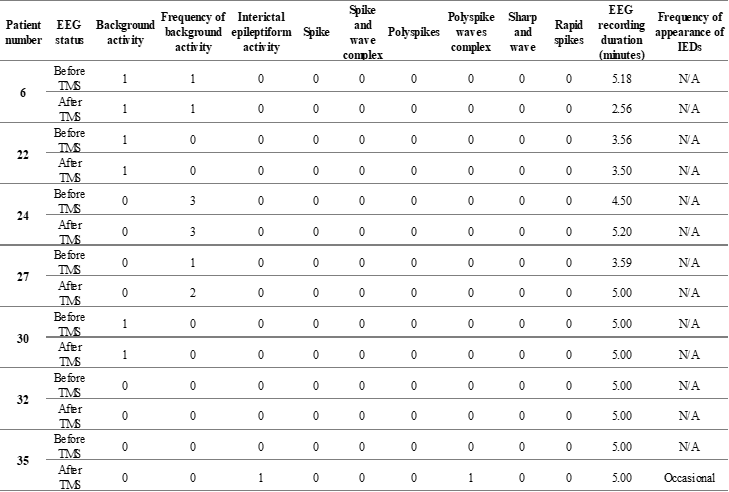


Background activity = 0 normal (posterior dominant rhythm alpha band), 1 abnormal (slow), 2 not assessable. Frequency of Background Activity = 0 alpha rhythm, 1 theta rhythm, 2 delta rhythm, 3 beta rhythm. Interictal epileptiform activity presence = 0 no, 1 yes. Spike = 0 no, 1 yes. Spike and wave complex = 0 no, 1 yes. Polyspikes = 0 no, 1 yes. Polyspike wave complex = 0 no, 1 yes. Sharp and wave complex = 0 no, 1 yes. Rapid spikes = 0 no, 1 yes. Frequency of appearance of IEDs (Interictal epileptiform discharges) = N/A not observed, Occasional appearance of IEDs between 1 event per minute to 1 event to per 5 minutes.

**Figure A1.** Resting HD-EEG recording showing 10-second epochs per page (patient #35). The EEG is displayed with 63-sensors, 200 μV sensitivity, transversal montage and common average. Panel 1 shows part of the recording before the TMS session. Panel 2 shows the trace after the TMS session.

1. According to the SCORE classification, this EEG trace was classified with alpha background rhythm posterior predominant and free from interictal epileptiform discharges.

1. This EEG trace was classified with alpha background rhythm posterior predominant and occasional interictal epileptiform discharges at the FT7 and T7 electrodes (red arrows), both away from the stimulation targets.

Figure A2. Resting HD-EEG recording showing 10-second epochs per page (healthy control). The EEG is displayed with 63-sensors, 200 μV sensitivity, transversal montage and common average. Panel 1 shows the trace before the TMS session. Panel 2 shows the trace after the TMS session.

1) Posterior alpha activity typical of healthy control recordings.

2) Example of trace with high number of artefacts present. This might be due to the electrodes having higher impedance values towards the end of the recording, and/or the participant becoming increasingly restless after undergoing the experiment for at least 2-3 hours. Electrode TP-10 was removed from analysis due to being artefactual.

# References

Beniczky S, Aurlien H, Brøgger JC, Hirsch LJ, Schomer DL, Trinka E, et al. Standardized computer-based organized reporting of EEG: SCORE – Second version. Clin Neurophysiol 2017;128:2334–46. <https://doi.org/10.1016/j.clinph.2017.07.418>
